# Effect of Alternate Wetting and Drying Irrigation on the Nutritional Qualities of Milled Rice

**DOI:** 10.3389/fpls.2021.721160

**Published:** 2021-09-09

**Authors:** Tao Song, Debatosh Das, Fuyuan Zhu, Xiaofeng Chen, Moxian Chen, Feng Yang, Jianhua Zhang

**Affiliations:** ^1^Co-Innovation Center for Sustainable Forestry in Southern China, College of Biology and the Environment, Nanjing Forestry University, Nanjing, China; ^2^Shenzhen Research Institute, The Chinese University of Hong Kong, Shenzhen, China; ^3^Guangdong Provincal Key Laboratory of Seed and Seedling Health Management Technology, Shenzhen Noposion Agrochemical Co., Ltd, Shenzhen, China; ^4^School of Life Sciences and State Key Laboratory of Agrobiotechnology, The Chinese University of Hong Kong, Hong Kong, SAR China; ^5^Department of Biology, Hong Kong Baptist University, Kowloon, Hong Kong, SAR China

**Keywords:** rice, irrigation, nutrition, metabolome, lipids, phenolic acids, alkaloids, amino acids

## Abstract

Alternate wetting and drying (AWD) irrigation has been widely used to save irrigation water during rice production when compared to the traditionally continuous flooding (CF). Although the influence of AWD on water-saving potential and grain yield has been studied before, its detailed effect on grain nutritional quality in milled rice remains relatively unexplored. In this study, AWD could maintain grain yield as compared with CF. Thus, we undertook efforts to compare the nutritional traits of milled rice irrigated with AWD and CF regimes. A targeted metabolome assay on milled rice identified 74 differentially accumulated metabolites (DAMs) with 22 up- and 52 down-accumulated metabolites under AWD vs. CF. Clustering of the metabolite content obtained in this assay suggested that most of the metabolites showing significant changes belonged to “lipids,” “alkaloids,” and “phenolic acids.” In addition, total protein, starch, lipid, and amino acids content were measured to correlate it with the differential accumulation of specific metabolites detected in the metabolome. Overall, the data suggested that AWD may improve the nutritional performance of milled rice by increasing amino acids and phenolic acids and decreasing lipids and alkaloids. Our study provides research proof for the need for the optimization of irrigation to optimize rice nutritional qualities.

## Introduction

Metabolites play an important role in plant growth, plant–environment interactions, and human nutrition (Saito and Matsuda, 2010; Wurtzel and Kutchan, 2016). Metabolite analysis serves as a reliable and detailed measure of food nutritional quality under different agricultural practices (such as irrigation regime and fertilizer application). Targeted metabolome provides a biased approach to quantify only a specified group of metabolites for such food metabolite analysis (Ramautar et al., 2006; Monton and Soga, 2007). Rice (*Oryza sativa L*.) is one of the most important staple crops in the world, in terms of production area and global consumption. Rice contribution to global food energy, protein, and fat needs is 21, 14, and 2%, respectively (Vlachos and Arvanitoyannis, 2008). In China, more than 95% of rice is irrigated with continuous flooding (CF) irrigation regime, which consumes a lot of manpower and material and financial resources (Mahajan et al., 2012). Lately, as an alternative water-saving practice, the alternate wetting and drying (AWD) irrigation regime has been adopted in many Asian countries including China, Bangladesh, India, and Vietnam. Importantly, AWD irrigation can reduce water consumption by 30–35% (Zhang et al., 2009). Subsequently, many studies were conducted on the effects of AWD on rice (Cheng et al., 2003; Norton et al., 2017a; Graham-Acquaah et al., 2019; Song et al., 2019; Dossou-Yovo and Saito, 2021). For example, a field study suggested that the grain yield increased under AWD vs. CF, and the rice nutritional quality also increased with lower content of sulfur, calcium, iron, and arsenic, and higher contents of manganese, copper, and cadmium (Norton et al., 2017a). However, some researchers found that AWD resulted in negative effects on rice grain quality with regard to an increase in chalkiness (Graham-Acquaah et al., 2019). Thus, we hypothesized that the metabolite content of milled rice will be significantly affected with differences in irrigation regime depending upon the quantity of water used. Hence, we compared differences in metabolite levels for milled rice grown under AWD and CF using widely targeted UPLC–ESI-MS/MS-based metabolome assay. The results of this study would provide hints for rice production practices to obtain milled rice of improved quality.

## Methods

### Plant Materials and Growth Conditions

In this study, Japonica rice Nipponbare was used and the experiment was conducted in the greenhouse of the Shenzhen Research Institute, Chinese University of Hong Kong, Shenzhen, Guangdong, China (22° 32 ′N, 113° 56 ′E). Artificial light was not used in the greenhouse. The temperature, sunlight, and humidity of the greenhouse used in the experiment have been shown in Supplementary Figure 1. The soil properties were as follows: pH 4.91 (soil water ratio 1:5), total nitrogen 1.73 g·kg^−1^, total phosphorus 0.72 g·kg^−1^, total potassium 29.3 g·kg^−1^, organic carbon 22.5 g·kg^−1^, alkali hydrolysable nitrogen 216 mg·kg^−1^, Olsen phosphorus 36.7 mg·kg^−1^, and available potassium 115.0 mg·kg^−1^. Each polyvinyl chloride (PVC) pot with a height of 30 cm and diameter of 34 cm was filled with 15 kg soil, and 6 seedlings were planted in each pot. The pot experiment was carried out in a completely randomized design. The application of base fertilizer in each pot was as follows: urea 1 g, phosphorus pentoxide 1.2 g and potassium oxide 0.9 g. Further addition of urea (0.8 g) was applied to each pot at tillering and early heading stages, respectively. Four-week-old seedlings were transplanted to pots on April 28. Continuous flooding and AWD irrigation were commenced 2 weeks after seedling transplantation to pots, and plants were irrigated with tap water (turbidity < 0.12 NTU, pH = 7.0, free chlorine = 0.6 mg/L). Under CF irrigation, a 2–5 cm water layer was always maintained in the basin. Under AWD irrigation, water was added to the pot until restore the water layer of 2–3 cm when the soil water potential of 15–20 cm soil layer reached −15 kPa (measured by tensiometer). The heading date of AWD and CF treatments was between June 18 and 26, and the maturation date was July 30. The whole growth period was 121 days in both the treatments.

### Measurement of Grain Yield and Yield Components

Grain yield was measured at the maturity stage. First, the grains were dried in the sun for 3 days (29–35°C), and then, grain yield was measured. Yield components such as panicle number per plant, spikelet number per panicle, grain-filling percentage, and 1,000-grain weight were determined from 12 plants as one biological replicate (three biological replicates were conducted). Data were subjected to ANOVA in IBM, USA SPSS Statistics ver. 18 (IBM, USA) and compared *via post hoc* honest significant difference test of Turkey. The data are presented as mean (*n* = 3) ± SEs, and unshared letters indicate a significant difference at *P* = 0.05.

### Sample Collection and Extraction for Metabolic Analysis

In the mature stage, grains from 12 rice plants were collected to make a biological replicate, and such three biological replicates were conducted. Samples were dried in the sun for 3 days and stored at room temperature for 3 months, and then, the samples were processed with a small rice milling machine (2011 type, Shaoxing, China). Biological samples were freeze-dried in a vacuum freeze-drier (Scientz-100F, China). The freeze-dried samples were crushed using a mixer mill (MM 400, Retsch, Germany) with a zirconia bead for a total of 1.5 min at 30 Hz frequency. Approximately 100 mg of lyophilized powder was dissolved in 1.2 mL of 70% methanol, vortexed for 30 s every 30 min for six times in total, and then incubated in a refrigerator at 4°C overnight. The next day, samples were centrifuged at 12,000 rpm for 10 min, and the extracts were filtered with a filter (SCAA-104, 0.22 μm pore size; ANPEL, China, http://www.anpel.com.cn/) before being put into the ultra performance liquid chromatography (UPLC) workflow.

### UPLC–MS Workflow

The sample extracts obtained earlier were analyzed using a UPLC–ESI-MS/MS system (UPLC, SHIMADZU Nexera X2, www.shimadzu.com.cn/; MS, Applied Biosystems 4500 Q TRAP, www.appliedbiosystems.com.cn/). The analytical conditions were as follows: (a) UPLC column, Agilent SB-C18 (1.8 μm, 2.1 mm^*^100 mm); (b) mobile phase with solvent A containing pure water with 0.1% formic acid and solvent B containing acetonitrile with 0.1% formic acid. Sample measurements were performed with a gradient program that employed the starting conditions of 95% A, 5% B. Within 9 min, a linear gradient toward 5% A, 95% B was programmed, and a composition of 5% A, 95% B was kept for 1 min. Subsequently, a composition of 95% A, 5.0% B was adjusted within 1.10 min and kept for 2.9 min. The flow velocity was set as 0.35 mL per min. The column oven was set at a temperature of 40°C. The injection volume was 4 μl. Subsequently, the resulting effluent was input to an ESI-triple quadrupole-linear ion trap (Q TRAP)-MS. The effluent was alternatively connected to an ESI-triple quadrupole-linear ion trap (Q TRAP)-MS.

### ESI-Q TRAP-MS/MS

On the AB4500 Q TRAP MS system (with ESI turbo in-spray interface), operating in both the negative and positive modes as controlled by Analyst 1.6.3 (AB Sciex, US), both linear ion trap (LIT) and triple quadrupole (QQQ) mode scans were obtained. This operation was conducted with the following parameter: (a) turbo spray as ion source; (b) 550°C as source temperature; (c) 4500 volts in negative ion mode/5500 volts in positive ion mode as ion spray voltage; (d) gas I (GSI), gas II (GSII) and curtain gas (CUR) at 50, 60, 25 psi pressure; (e) high collision-activated dissociation.

In QQQ and LIT modes, 10 and 100 μmol/L polypropylene glycol solutions were used to perform instrument tuning and mass calibration. In addition, QQQ scans were obtained with multiple reaction monitoring using nitrogen (collision gas) set to medium. For each period of metabolite elution, a specific set of MRM transitions were monitored according to the metabolite nature. Declustering potential (DP) and collision energy (CE) were optimized accordingly. Retention times were recorded accordingly.

### Metabolite Detection and Quantification

Based on the metabolite information contained in the Metware database MWDB (Metware Biotechnology Co., Ltd., Wuhan, China) and the public database (MassBank, http://www.massbank.jp), primary and secondary MS data were subjected to qualitative analyses (Yang et al., 2019). Isotope signal, repeated signals such as K^+^, Na^+^, ${\text{NH}}_{4}^{+}$, and fragments from high molecular weight metabolites were excluded in the analysis. Metabolite quantification was carried out *via* the MRM mode of the QQQ mass spectrometer. In the MRM mode, the quadrupole first searched for precursor ions of target substances while screening for any ions derived from substances of different molecular weights to eliminate their interference preliminarily. The precursor ions were fragmented *via* induced ionization in the collision chamber to form many fragment ions, which were then filtered through QQQ to select single-fragment ions with the desired characteristics while eliminating the interference from non-target ions. This step leads to increased precision and repeatability of the quantification results. In the MS data, all the mass spectrum peaks were subjected to area integration. To compare the differences in the content of each detected metabolite from different samples, the metabolite mass spectral peaks were corrected depending upon prior information on metabolite retention time (RT) and peak type, which ensured the accuracy of the qualitative and quantitative analyses.

### Quantification of Total Proteins, Starch, Free Amino Acid, and Lipids

Total protein was measured with Kjeldahl nitrogen determination methods as mentioned in Wu et al. (2020). Milled rice sample powder of 0.2 g, 1.0 g of catalyst (CuSO_4_:Na_2_SO_4_ = 1:10), and 4.0 mL of H_2_SO_4_ were added into a digestion tube (100 mL) in turn. The mixture was heated at 420°C for 2 h in a digestion stove immediately. The mixture was cooled to room temperature after digestion. Then, 10 mL ddH_2_O was added into the digestion tube. The mixed solution was analyzed using a Kjeltec 8400 Autoanalyzer (Foss, Sweden). Protein content of the rice flour was calculated from the total N content by multiplying a conversion factor of 5.95.

Starch content was obtained by a method previously mentioned (de Souza et al., 2016). Milled rice powder of 5 g was extracted with 75 mL of 0.18% NaOH at 30°C for 30 min. Then, the mixture was centrifuged at 3,380 g for 5 min for separating the starch-rice. The resulting starch-rice part was neutralized to pH 7.0 by acidification with 0.1M HCl. The resultant starch was dried at 30°C for 36 h to obtain a quantifiable constant weight.

For free amino acid determination, the samples were subjected to the ninhydrin determination method (Lin et al., 2010). Briefly, 0.5 g of milled rice powder samples were extracted with 5 mL of 10% acetic acid, filled to 100 mL with a 0.2 M acetate buffer, filtration of crude extracts using filter paper. The standard sample used was lysine in gradient concentrations mixed with ninhydrin colorimetric solution along with experimental samples consisting of the aforementioned filtered samples mixed with ninhydrin colorimetric solution. Spectrometry values were obtained for standard and experimental samples at a wavelength of 580 nm. Total amino acid content was quantified based on the lysine standard curve.

Total lipids were extracted with the Soxhlet extraction methods (Castro and Garcia-Ayuso, 1998). A milled rice sample of 0.5 g was mixed with 210 mL chloroform/methanol (2:1, v/v) for 24 h for extraction of lipids. Subsequently, the lipids were washed with 37.5 mL saltwater (0.7–0.75% NaCl). From this, the solvent layer was separated by using a separation funnel. To precipitate, evaporation was carried out in a rotary vacuum evaporator. The remaining residuals were resuspended in a small amount of diethyl ether/hexane (1:1, v/v) solution and transferred into a preweighed vial for gravimetric determination.

Data were subjected to ANOVA, the data are presented as mean (*n* = 3) ± SEs, and unshared letters indicate significant difference at *P* = 0.05.

### Principal Component Analysis for Metabolic Profiling

An unsupervised principal component analysis (PCA) was performed with statistics function prcomp() within the R software environment (www.r-project.org). The input data were unit variance which was scaled before performing unsupervised PCA.

### Differential Metabolite Analysis

Values of variable importance in projection (VIP) were extracted from OPLS-DA output, which also contains score and permutation plots. Significantly accumulated metabolites in the comparison of AWD vs. CF were determined using a cut-off of importance in projection VIP ≥ 1 and *P* ≤ 0.05. Plots were generated using the R package MetaboAnalystR (Chong and Xia, 2018). The data were log transformed (log_2_) and centered around the mean before OPLS-DA analysis. In order to avoid overfitting, a permutation test (200 permutations) was performed.

### KEGG Annotation and Enrichment Analysis

Identified metabolites were annotated using the KEGG compound database (http://www.kegg.jp/kegg/compound/). Annotated metabolites were then mapped to the KEGG pathway database (http://www.kegg.jp/kegg/pathway.html). Pathways that mapped to DAMs were then fed into metabolite sets enrichment analysis, and enrichment significance was determined with a hypergeometric test.

## Results

### Grain Yield and Yield Components

In order to investigate the effect of irrigation regimes on rice yield, both the grain yield and yield components under AWD and CF were quantified. There was no significant difference in grain yield, panicles per plant, spikelets per panicle, filled grains, and 1,000-grain weight between AWD and CF (Table 1).

**Table 1:** Grain yields and yield components under the two irrigation regimes.

**Irrigation regime**	**Grain yield (g/pot)**	**Panicles per plant**	**Spikelets per panicle**	**Filled grains (%)**	**1000-grain weight (g)**
CF	28.5 ± 1.21a	8.3 ± 0.58a	60.3 ± 4.93a	83.3 ± 1.45a	23.2 ± 0.33a
AWD	29.6 ± 0.87a	8.0 ± 1a	61.0 ± 4.58a	85.7 ± 1.75a	23.4 ± 0.29a

*Continuous flooding and alternate wetting and drying represent continuously flooded and alternate wetting and drying irrigation, respectively. Different letters indicate statistical significance at the P = 0.05 level within the same column, value are means (n = 3)*.

### Metabolome Assay of Milled Rice From AWD and CF

**Figure 1:**
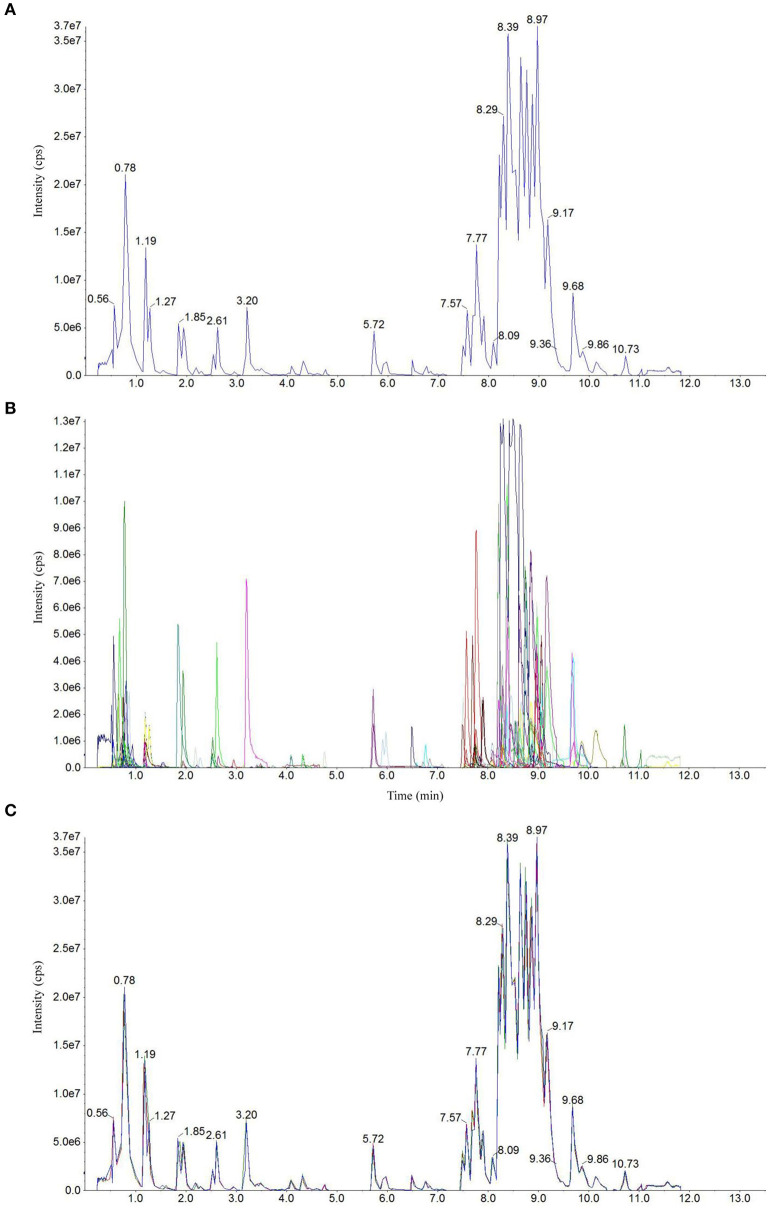
Detection of metabolites in UPLC–ESI-MS/MS. Total ion chromatographs of one quality control (QC) sample by mass spectrometry detection **(A)**, multipeak detection plot of metabolites in the multiple reaction monitoring (MRM) mode **(B)**, and overlapping map by QC sample **(C)**.

Total ion current (TIC) chromatogram plots of quality control (QC) samples as shown in Figure 1A depict a continuous representation of the intensity sum of all ions at different sampling time points in the shown mass spectrum. Figure 1B shows the multipeak detection plot of metabolites in MRM mode depicting the ion current plot of multiple substances, where the x-axis indicates RT of the metabolites and the y-axis indicates the ion current intensity measured in counts per second (cps). Based on the local metabolite database (Yan et al., 2019), qualitative and quantitative mass spectrometry analyses were conducted. Figure 1B revealed the constituent substances detected in the input samples. Each of the detected metabolites is represented with a different color in this plot. Further annotation and quantification information (such as metabolite names, classes, VIP values and *P* values) for all the detected metabolites is provided in Supplementary Dataset 1.

Technical repeatability (reproducibility of metabolite extractions and detections) was assessed by overlaying the TIC plots of different replicates of different QC samples (Figure 1C). The figure shows a significant overlap of the TIC plots of metabolites between the first and last QC samples, indicating high repeatability. Therefore, RT and peak intensities are consistent between these two QC samples, suggesting good signal stability during the detection of the same sample at different running times. The plots of the CF + AWD samples largely overlapped with each other suggesting good instrumental stability. Overall, it suggests that the data from our MS measurements showed good repeatability.

### Multivariate Analysis of MS-Detected Metabolites

**Figure 2:**
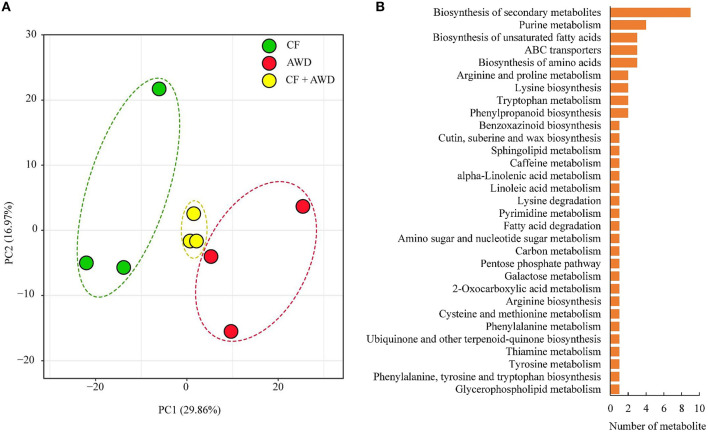
Exploratory analysis of metabolite accumulation in AWD vs. CF. **(A)** PCA analysis of metabolites identified in CF, AWD samples. Equal volumes of CF and AWD milled rice samples were mixed for use as a quality control (QC) sample. **(B)** Kyoto encyclopedia of genes and genomes classification of differentially accumulated metabolites.

Principal component analysis was performed on a total of 601 metabolites identified from both AWD and CF samples, and the results suggested close clustering, and good reproducibility of replicates within CF, AWD, and samples of CF + AWD and also distinct metabolome profiles of CF and AWD samples (Figure 2A). For differential metabolite accumulation analysis, we selected metabolites with *P* ≤ 0.05 in an AWD vs. CF comparison. These metabolites were further selected using a VIP cut-off value ≥ 1 in the orthogonal projections to latent structures-discriminate analysis (OPLS-DA) model. This led to the identification of 74 DAMs in the AWD vs. CF comparison (Supplementary Dataset 1). of these 74 metabolites, 52 were down-regulated and 22 were up-regulated in AWD. Mapping these 74 DAMs to KEGG metabolic pathway maps suggested significant enrichment of pathways belonging to “biosynthesis of secondary metabolites,” “purine metabolism,” and “biosynthesis of unsaturated fatty acids, biosynthesis of amino acids, ABC transporters” (Figure 2B).

### Classification Clustering of Differentially Accumulated Metabolites

**Figure 3:**
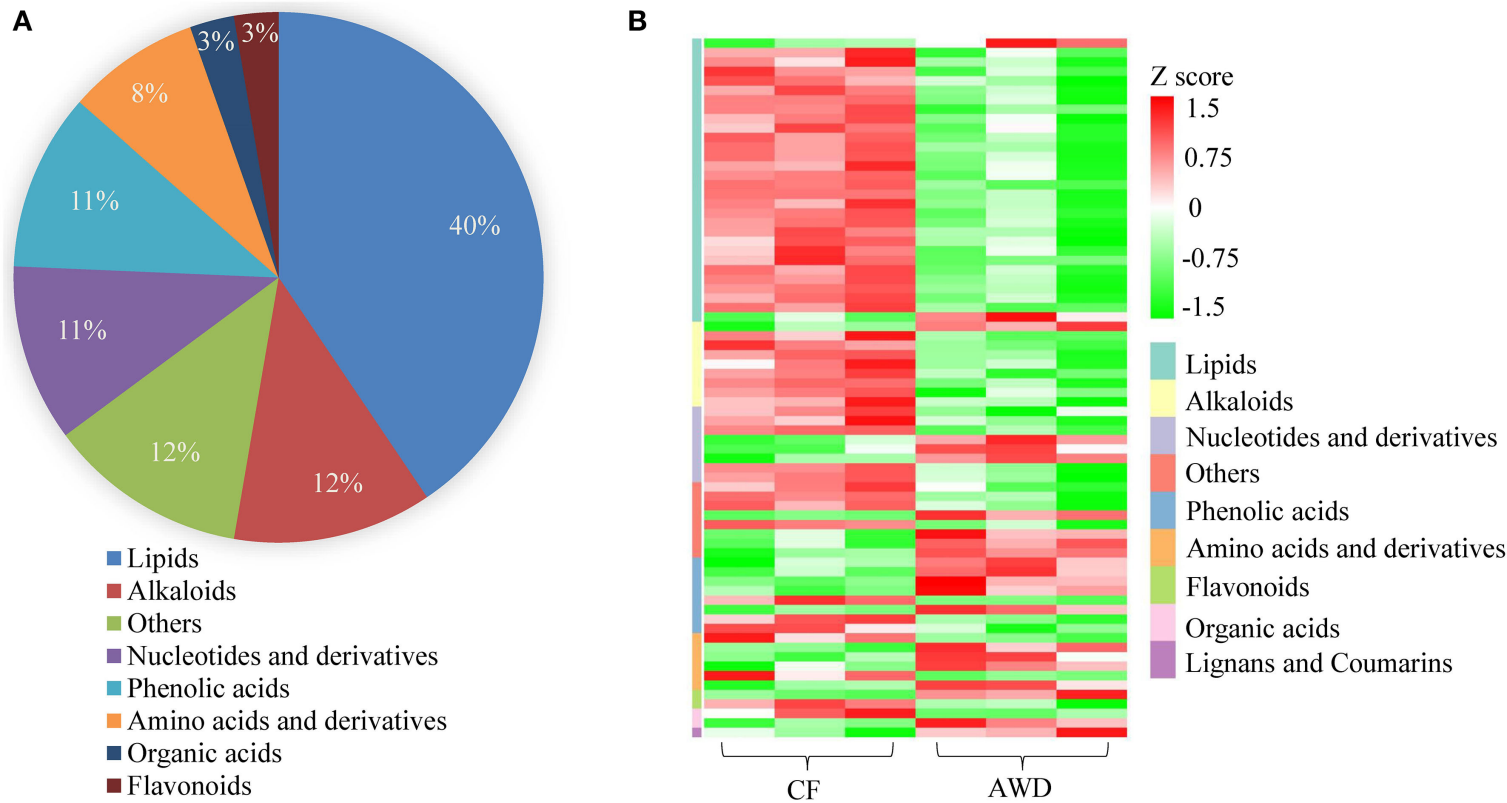
Preliminary characterization of differentially accumulated metabolites in AWD vs. CF. **(A)** Pie chart depicting the biochemical categories of the differential metabolites identified between CF and AWD samples. **(B)** Cluster analysis of metabolites from samples of CF and AWD. The color indicates the level of accumulation of each metabolite, from low (green) to high (red). The Z-score represents the deviation from the mean by SD units.

**Figure 4:**
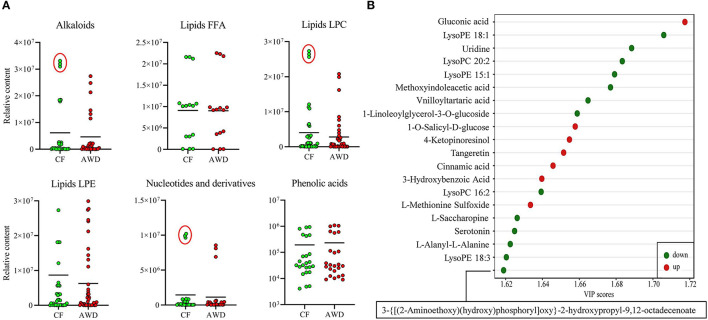
Relative contents of differentially accumulated metabolites in AWD vs. CF. **(A)** Relative content values of upregulated or downregulated metabolites were plotted for the metabolite groups that showed distinct clustering pattern in Figure 3A hierarchical clustering such as alkaloids, lipids, nucleotides, and phenolic acids between AWD and CF irrigation types. **(B)** Different metabolites in OPLS-DA model with top 20 VIP values in AWD vs. CF comparison.

**Figure 5:**
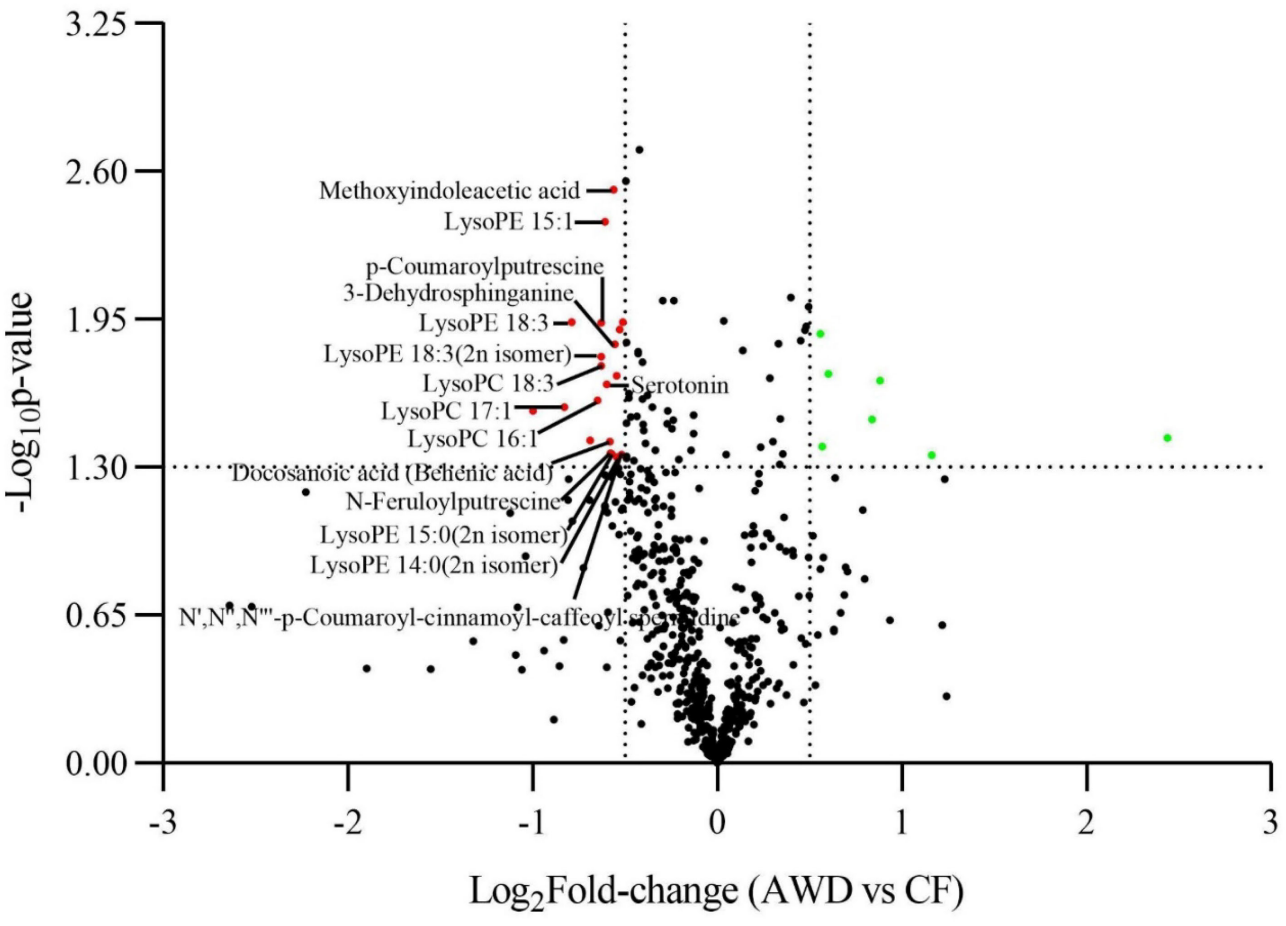
Volcano plot comparison of differentially accumulated metabolites in AWD vs. CF. A plot correlating log_10_ *P*-value with log_2_FC (AWD vs. CF) displaying metabolites up-/down-regulated in the metabolome assay.

For hierarchical clustering, data transformation based on the log_10_ scale was performed on peak area values for each metabolite in order to normalize the data and eliminate the confounding effects of metabolite quantity in its pattern recognition. The earlier identified 74 DAMs were categorized into eight different classes (Figure 3A). Differentially accumulated metabolites consisted of lipids (30 in total), alkaloids (nine in total), nucleotides and derivatives (eight in total), phenolic acids (eight in total), amino acids and derivatives (six in total), organic acids (two in total), flavonoids (two in total), and others (nine in total). This analysis revealed mainly two distinct cluster groups in the AWD vs. CF comparison (Figures 3B, 4A). Those showing relatively lower accumulation in AWD vs. CF irrigation belonged to “lipids” and “alkaloids” and for the majority of DAMs to “nucleotides and derivatives.” An opposite trend was observed for the majority of DAMs belonging to “phenolic acids” and “amino acids and derivatives.” In an overview of constituent DAM metabolites in these groups, Figure 4B shows the distribution of different metabolites with top 20 VIP values in the OPLS-DA model in AWD vs. CF comparison. These 20 metabolites consisted of seven lipids, three alkaloids, and three phenolic acids (the top three classes). In addition, a volcano plot of -log_10_(*P* value) vs. log_2_FC (in AWD vs. CF), revealed that metabolites belonging to alkaloid and lipids metabolites were significantly down-regulated to a higher extent (Figure 5). Among the highly down-regulated metabolites included LysoPC 17:1 and LysoPE 18:3.

### Quantitative Comparison of Total Protein, Starch, Free Amino Acid, and Lipids Content in Milled Rice

Protein and starch content in the milled rice were similar between AWD and CF irrigation (Table 2). The protein content of milled rice ranged between 8.43 and 8.64%, and starch content ranged between 83.18 and 84.56% in the two irrigation regimes. Free amino acid content was higher in AWD (12.55 mg/100g) when compared to CF (11.40 mg/100 g). On the contrary, total lipids content was lower in AWD (1.51%) as compared to CF (1.82%).

**Table 2:** Total protein, starch, free amino acid, and lipids content of milled rice.

**Irrigation regime**	**Total protein (%)**	**Starch (%)**	**Free amino acid (mg/100g)**	**Lipids (%)**
CF	8.64 ± 0.65a	83.18 ± 1.57a	11.40 ± 0.14b	1.82 ± 0.15a
AWD	8.43 ± 0.29a	84.56 ± 2.04a	12.55 ± 0.23a	1.51 ± 0.12b

*CF and AWD represent continuously flooded and alternate wetting and drying irrigation, respectively. Different letters indicate statistical significance at the P = 0.05 level within the same column, value are means (n = 3)*.

## Discussion

Previous studies have provided contrasting conclusions about the effect of irrigation regimes on agronomic traits. For example, it was found that AWD (where irrigation was applied to fields when the soil water potential reached −25 kPa at a depth of 15–20 cm) increased the grain filling and grain weight of inferior spikelets (by ~12%) in the Huaidao 9 cultivar, which finally contributed to a 10% increase in grain yield (Zhang et al., 2010). However, other researchers had found that AWD (where irrigation was applied when pond water disappeared in the PVC tubes to reflood the field up to a depth of 5 cm) had no effects on the grain yield of Yangliangyou 6 cultivar (Yao et al., 2012). In another study, AWD (where IR66 cultivars) were watered every 8 days (up to a depth of 5–7 cm) even reduced the grain yield by 19–26% (Tabbal et al., 2002). In this study, the agronomic traits of rice crops irrigated with AWD and CF showed no significant difference between each other (Table 1). All these differences in observations may be due to differences in experimental conditions and rice varieties used in the respective studies (Peng and Bouman, 2007; Song et al., 2018).

Milled rice consists of endosperm without embryo and bran layers. Its quality is determined mainly by endosperm nutrient composition, which involves around 70~80% starch, 7–10% proteins, and about 1% lipids (Jaksomsak et al., 2020). In our study, we use the UPLC/ESI-Q TRAP-MS/MS to analyze the metabolome profiling of AWD and CF rice plants to evaluate the impact of AWD on milled rice quality.

Most down-accumulated metabolites belonged to lipids. Lipids content and proportion of different types of lipids in rice play a minor but still physiologically and functionally significant role in human nutrition since it is consumed in large amounts by half of the world population on a daily basis (Vega-Gálvez et al., 2010). Lipids comprise of three major types of lipids, which are lysophosphatidylcholine (LPC), lysophosphatidylethanolamine (LPE), and free fatty acids (Tong and Bao, 2019). In this study, most of these down-accumulated lipids belonged to the LPC and LPE categories (Figures 4, 5). Decreased total lipids (Table 2) were consistent with the finding of our metabolome assay (Figure 3, Table 3). Among these lipids, α-linolenic acid plays an important role in biotic and abiotic stress (Tan et al., 2021). It was found that acyl-CoA-binding protein 2 (ACBP2) can bind to LPC to promote its degradation in response to cadmium-induced oxidative stress in *Arabidopsis* (Gao et al., 2010). In this study, both α-linolenic and LPC showed a decrease in response to AWD.

**Table 3:** Differentially accumulating metabolites in AWD vs. CF comparison.

**Compounds**	**Class**	**VIP**	***P*_value**	**Type**
Palmitaldehyde	Lipids	1.48	0.044	up
1-Linolenoyl-rac-glycerol-diglucoside	Lipids	1.50	0.039	up
γ-Linolenic Acid	Lipids	1.53	0.030	down
α-Linolenic Acid	Lipids	1.49	0.036	down
3-Dehydrosphinganine	Lipids	1.61	0.014	down
Docosanoic acid	Lipids	1.46	0.039	down
LysoPC 10:0	Lipids	1.55	0.024	down
LysoPE 14:0	Lipids	1.55	0.045	down
LysoPE 15:1	Lipids	1.68	0.004	down
LysoPC 12:0	Lipids	1.46	0.047	down
LysoPE 15:0	Lipids	1.46	0.044	down
LysoPE 18:3	Lipids	1.62	0.012	down
LysoPE 18:3	Lipids	1.59	0.016	down
LysoPE 18:2	Lipids	1.59	0.027	down
LysoPE 18:2	Lipids	1.51	0.033	down
LysoPC 15:1	Lipids	1.56	0.046	down
LysoPE 18:1	Lipids	1.71	0.003	down
LysoPE 18:1	Lipids	1.61	0.032	down
LysoPC 16:2	Lipids	1.57	0.016	down
LysoPC 16:2	Lipids	1.64	0.017	down
LysoPC 16:1	Lipids	1.56	0.025	down
LysoPC 17:2	Lipids	1.54	0.025	down
LysoPE 20:2	Lipids	1.40	0.045	down
LysoPC 17:1	Lipids	1.48	0.027	down
1-Linoleoylglycerol-3-O-glucoside	Lipids	1.66	0.020	down
LysoPC 18:3	Lipids	1.61	0.014	down
LysoPC 18:3	Lipids	1.58	0.018	down
LysoPC 18:1	Lipids	1.58	0.024	down
LysoPC 19:0	Lipids	1.58	0.016	down
LysoPC 20:2	Lipids	1.68	0.002	down
Diethanolamine	Alkaloids	1.62	0.015	up
Serotonin	Alkaloids	1.62	0.022	down
Methoxyindoleacetic acid	Alkaloids	1.68	0.003	down
p-Coumaroylputrescine	Alkaloids	1.59	0.012	down
N-Feruloylputrescine	Alkaloids	1.43	0.044	down
3-[(2-Aminoethoxy)(hydroxy)phosphoryl]oxy-2-hydroxypropyl-9,12-octadecenoate	Alkaloids	1.62	0.025	down
Ergotamine	Alkaloids	1.55	0.040	down
N′,N″N^‴^-p-Coumaroyl-cinnamoyl-caffeoyl spermidine	Alkaloids	1.41	0.044	down
4-Hydroxybenzaldehyde	Phenolic acids	1.50	0.041	up
Tyrosol	Phenolic acids	1.60	0.012	up
Cinnamic acid	Phenolic acids	1.65	0.044	up
Sinapinaldehyde	Phenolic acids	1.56	0.031	up
1-O-Salicyl-D-glucose	Phenolic acids	1.66	0.009	up
Vnilloyltartaric acid	Phenolic acids	1.66	0.012	down
1,3-O-Diferuloylglycerol	Phenolic acids	1.58	0.012	down
Di-O-Glucosylquinic acid	Phenolic acids	1.44	0.030	down
L-Alanyl-L-Alanine	Amino acids and derivatives	1.62	0.028	down
L-Methionine Sulfoxide	Amino acids and derivatives	1.63	0.013	up
N-α-Acetyl-L-ornithine	Amino acids and derivatives	1.49	0.044	up
2,6-Diaminooimelic acid	Amino acids and derivatives	1.50	0.041	up
L-Saccharopine	Amino acids and derivatives	1.63	0.042	down
L-Aspartic acid-O-diglucoside	Amino acids and derivatives	1.56	0.020	up

Alkaloids also show down-accumulation in AWD vs. CF-irrigated milled rice. These comprise of secondary metabolites, which have both ring and non-ring structures and contain nitrogen atoms in a negative oxidation state (Kaur and Arora, 2015). Like lipids, alkaloid composition in seeds can be significantly altered by environmental conditions, especially in the vegetative growth stages, for example, in *Lupinus angustifolius L*. (Christiansen et al., 1997). Among these alkaloids, ergotamine belongs to ergot alkaloids and regular intake of cereals with these alkaloids may result in ergotism characterized by loss of toes and fingers and sometimes may prove fatal (Schummer et al., 2020). Serotonin (mammalian neurotransmitter) biosynthesis is induced in rice in response to insect infestation, while its suppression provides resistance to rice pests such as plant hoppers and stem borers (Lu et al., 2018). The AWD plants may show a different resistance to these pests. In this study, alkaloid compounds were less accumulated in AWD samples suggesting that AWD may not only make the rice safer to people but also to the destructive pests, which are worth attention.

Plant phenolic acids are a group of polyphenols that occur naturally inside the plants and serve multiple benefits to plants and as a dietary component. First, these are potent chemicals providing resistance to pathogens mainly pathogenic fungi (Mandal et al., 2010). For example, cinnamic acid is induced by beneficial Rhizobia bacteria in rice and is implicated in resistance to Rhizoctonia (Mishra et al., 2006). Another polyphenol called tyrosyl was shown to have very potent antifungal activity not only in plant diseases but also in human infections (Abdel-Rhman et al., 2020; Berne et al., 2020). Sinapinaldehyde or Sinapyl aldehyde is another class of antifungal compound produced naturally in plants. Second, plant phenolics are implicated in plant growth regulation. For example, cis-cinnamic acid was shown to be a natural plant growth-promoting compound (Steenackers et al., 2019). Environmental changes such as the onset of drought stress enhance phenolic acids in both *Amaranthus tricolor* and rice leaves (Quan et al., 2016). Third, these compounds serve nutritional benefits in the human diet. For example, 4-hydroxybenzaldehyde has been long used in Chinese medicine for treating migraines and nervous disorders and has also been associated with acute wound healing (Kang et al., 2017). In this study, most of the phenolic acids detected in our metabolome assay accumulated more in AWD vs. CF, indicating that AWD may help to improve plant resistance to pathogens and increase the antioxidant composition of milled rice making it more nutritious.

Most of the amino acids and derivatives (4 of 6) up-accumulated in AWD vs. CF (Figure 3 and Supplementary Dataset 1). Amino acids are utilized for the synthesis of storage proteins, as a precursor in the biosynthesis of other metabolites and as an energy source (Amir et al., 2018). The observed increase in free amino acid (Table 2) was consistent with the output of metabolome analysis (Figure 3 and Supplementary Dataset 1). Starch and protein are the highest abundant compounds in milled rice, but there was no significant difference in total starch and protein content in AWD vs. CF grown milled rice, indicating that AWD did not negatively alter the levels of basic nutritional components of the milled rice. Since the biomass response and elemental concentrations in 22 different rice cultivars treated with AWD were found to be similar (Norton et al., 2017b), it can be assumed that the major observed changes in metabolite accumulation in this study were genotype-independent and mainly depended on irrigation regime (AWD vs. CF) and the water quantity used.

## Conclusion

The data suggest an improvement in milled rice quality when irrigated with AWD *via* an increase in phenolic acids and amino acids while lowering lipids and alkaloids. Thus, AWD improves health-benefitting nutrients in milled rice and should be preferred over CF. This calls for more use of AWD in rice irrigation to not only benefit from its water-saving trait but also because it may significantly improve the nutritional quality of milled rice that can be better marketed and thus increase sales and income of the farmers. In addition, this study calls out for a deeper investigation of all nutritional components in rice and other crops based on the volume and periodicity of water input.

## Data Availability Statement

The original contributions presented in the study are included in the article/Supplementary Material, further inquiries can be directed to the corresponding authors.

## Author Contributions

TS, DD, and FY conducted the experiments. JZ and FY organized and supervised the overall project. TS, DD, and XC performed the data analysis and wrote the manuscript. JZ edited the manuscript. FZ and MC critically commented on the manuscript. All authors contributed to the article and approved the submitted version.

## Funding

This study was supported by the Natural Science Foundation of Hunan Province (2021JJ40247), the Science Technology and Innovation Committee of Shenzhen (JSGG20170822153048662 and GJHZ20190821160401654), Platform funding for Guangdong Provincial Enterprise Key Laboratory of Seed and Seedling Health Management Technology (2021B1212050011), National Key Research and Development Program of China (2017YFE0118100), the Basic and Applied Basic Research Foundation of Guangdong Province (2020A1515110586), Jiangsu Agricultural Science and Technology Innovation Fund (CX(21)2023), and the Hong Kong Research Grant Council (14177617, 12103220, and AoE/M-403/16).

## Conflict of Interest

XC was employed by the company Shenzhen Noposion Agrochemical Co., Ltd. The remaining authors declare that the research was conducted in the absence of any commercial or financial relationships that could be construed as a potential conflict of interest.

## Publisher's Note

All claims expressed in this article are solely those of the authors and do not necessarily represent those of their affiliated organizations, or those of the publisher, the editors and the reviewers. Any product that may be evaluated in this article, or claim that may be made by its manufacturer, is not guaranteed or endorsed by the publisher.

## Supplementary Material

Supplementary Dataset 1 Overall metabolites detected in alternate wetting and drying and continuous flooding treated milled rice.

Supplementary Figure 1 Temperature, sunlight, and humidity of greenhouse.

## References

[B1] Abdel-Rhman S. H. Rizk D. E. Abdelmegeed E. S. (2020). Effect of sub-minimum inhibitory concentrations of tyrosol and EDTA on quorum sensing and virulence of *Pseudomonas aeruginosa*. Infect. Drug Resist. 13, 501–3511. 10.2147/IDR.S264805 33116669 PMC7550211

[B2] Amir R. Galili G. Cohen H. (2018). The metabolic roles of free amino acids during seed development. Plant Sci. 275, 11–18. 10.1016/j.plantsci.2018.06.011 30107877

[B3] Berne S. Kovaevi N. Kastelec D. Javornik B. Radisek S. (2020). Hop polyphenols in relation to verticillium wilt resistance and their antifungal activity. Plants 9:1318. 10.3390/plants9101318 33036218 PMC7601901

[B4] Castro M. Garcia-Ayuso L. E. (1998). Soxhlet extraction of solid materials: an outdated technique with a promising innovative future. Anal. Chim. Acta 369, 1–10. 10.1016/S0003-2670(98)00233-5

[B5] Cheng W. Zhang G. Zhao G. Yao H. Xu H. (2003). Variation in rice quality of different cultivars and grain positions as affected by water management. Field Crops Res. 80, 245–252. 10.1016/S0378-4290(02)00193-4

[B6] Chong J. Xia J. (2018). MetaboAnalystR: an R package for flexible and reproducible analysis of metabolomics data. Bioinformatics 34, 4313–4314. 10.1093/bioinformatics/bty528 29955821 PMC6289126

[B7] Christiansen J. Jørnsgård B. Buskov S. Olsen C. (1997). Effect of drought stress on content and composition of seed alkaloids in narrow-leafed lupin, *Lupinus angustifolius* L. Eur. J. Agron. 7, 307–314. 10.1016/S1161-0301(97)00017-8

[B8] de Souza D. Sbardelotto A. F. Ziegler D. R. Marczak L. D. F. Tessaro I. C. (2016). Characterization of rice starch and protein obtained by a fast alkaline extraction method. Food Chem. 191:36–44. 10.1016/j.foodchem.2015.03.032 26258699

[B9] Dossou-Yovo E. R. Saito K. (2021). Impact of management practices on weed infestation, water productivity, rice yield and grain quality in irrigated systems in Côte d'Ivoire. Field Crops Res. 270:108209. 10.1016/j.fcr.2021.108209

[B10] Gao W. Li H.-Y. Xiao S. Chye M.-L. (2010). Acyl-CoA-binding protein 2 binds lysophospholipase 2 and lysoPC to promote tolerance to cadmium-induced oxidative stress in transgenic Arabidopsis. Plant J. 62, 989–1003. 10.1111/j.1365-313X.2010.04209.x 20345607

[B11] Graham-Acquaah S. Siebenmorgen T. J. Reba M. L. Massey J. H. Mauromoustakos A. Adviento-Borbe A.. (2019). Impact of alternative irrigation practices on rice quality. Cereal Chem. 96, 815–823. 10.1002/cche.10182

[B12] Jaksomsak P. Rerkasem B. Prom-U-Thai C. (2020). Variation in nutritional quality of pigmented rice varieties under different water regimes. Plant Prod. Sci. 24, 244–255. 10.1080/1343943X.2020.1819164

[B13] Kang C. W. Han Y. E. Kim J. Oh J. H. Cho Y. H. Lee E. J. (2017). 4-Hydroxybenzaldehyde accelerates acute wound healing through activation of focal adhesion signalling in keratinocytes. Sci. Rep. 7:14192. 10.1038/s41598-017-14368-y 29079748 PMC5660242

[B14] Kaur R. Arora S. (2015). Alkaloids-important therapeutic secondary metabolites of plant origin. J. Crit. Rev. 2, 1–8. Available online at: https://tarjomefa.com/wp-content/uploads/2018/05/9114-English-TarjomeFa.pdf21554009

[B15] Lin X. Zhang L. Lei H. Zhang H. Ruan R. (2010). Effect of drying technologies on quality of green tea. Int. Agric. Eng. J. 19, 30–37. Available online at: http://image.sciencenet.cn/olddata/kexue.com.cn/upload/blog/file/2011/1/201119152733835634.pdf

[B16] Lu H. P. Luo T. Fu H. W. Wang L. Tan Y. Y. Huang J. Z.. (2018). Resistance of rice to insect pests mediated by suppression of serotonin biosynthesis. Nat. Plants 4, 338–344. 10.1038/s41477-018-0152-7 29735983

[B17] Mahajan G. Chauhan B. Timsina J. Singh P. Singh K. (2012). Crop performance and water-and nitrogen-use efficiencies in dry-seeded rice in response to irrigation and fertilizer amounts in northwest India. Field Crops Res. 134:59–70. 10.1016/j.fcr.2012.04.011

[B18] Mandal S. M. Chakraborty D. Dey S. (2010). Phenolic acids act as signaling molecules in plant-microbe symbioses. Plant Signal. Behav. 5, 359–368. 10.4161/psb.5.4.10871 20400851 PMC2958585

[B19] Mishra R. Singh R. K. Jaiswal H. K. Kumar V. Maurya S. (2006). Rhizobium-mediated induction of phenolics and plant growth promotion in rice (*Oryza sativa* L.). Curr. Microbiol. 52, 383–389. 10.1007/s00284-005-0296-3 16586021

[B20] Monton M. R. N. Soga T. (2007). Metabolome analysis by capillary electrophoresis–mass spectrometry. J. Chromatogr. A 1168, 237–246. 10.1016/j.chroma.2007.02.065 17376458

[B21] Norton G. J. Shafaei M. Travis A. J. Deacon C. M. Danku J. Pond D.. (2017a). Impact of alternate wetting and drying on rice physiology, grain production, and grain quality. Field Crops Res. 205, 1–13. 10.1016/j.fcr.2017.01.016

[B22] Norton G. J. Travis A. J. Danku J. M. Salt D. E. Hossain M. Islam M. R.. (2017b). Biomass and elemental concentrations of 22 rice cultivars grown under alternate wetting and drying conditions at three field sites in Bangladesh. Food Energy Secur. 6, 98–112. 10.1002/fes3.110 28979771 PMC5599981

[B23] Peng S. Bouman B. (2007). Prospects for genetic improvement to increase lowland rice yields with less water and nitrogen. Frontis 2007, 249–264. 10.1007/1-4020-5906-X_20

[B24] Quan N. T. Anh L. H. Khang D. T. Tuyen P. T. Toan N. P. Minh T. N.. (2016). Involvement of secondary metabolites in response to drought stress of rice (*Oryza sativa* L.). Agriculture 6:23. 10.3390/agriculture6020023

[B25] Ramautar R. Demirci A. de Jong G. J. (2006). Capillary electrophoresis in metabolomics. TrAC Trends Anal. Chem. 25, 455–466. 10.1016/j.trac.2006.02.004

[B26] Saito K. Matsuda F. (2010). Metabolomics for functional genomics, systems biology, and biotechnology. Annu. Rev. Plant Biol. 61, 463–489. 10.1146/annurev.arplant.043008.092035 19152489

[B27] Schummer C. Zandonella I. An V. N. Moris G. (2020). Epimerization of ergot alkaloids in feed. Heliyon 6:e04336. 10.1016/j.heliyon.2020.e04336 32637710 PMC7330613

[B28] Song T. Xu F. Yuan W. Chen M. Hu Q. Tian Y.. (2019). Combining alternate wetting and drying irrigation with reduced phosphorus fertilizer application reduces water use and promotes phosphorus use efficiency without yield loss in rice plants. Agric. Water Manag. 223:105686. 10.1016/j.agwat.2019.105686

[B29] Song T. Xu F. Yuan W. Zhang Y. Liu T. Chen M.. (2018). Comparison on physiological adaptation and phosphorus use efficiency of upland rice and lowland rice under alternate wetting and drying irrigation. Plant Growth Regul. 86, 195–210. 10.1007/s10725-018-0421-5

[B30] Steenackers W. Houari I. E. Baekelandt A. Witvrouw K. Vanholme B. (2019). cis-Cinnamic acid is a natural plant growth-promoting compound. J. Exp. Bot. 70, 6293–6304. 10.1093/jxb/erz392 31504728 PMC6859716

[B31] Tabbal D. F. Bouman B. Bhuiyan S. I. Sibayan E. B. Sattar M. A. (2002). On-farm strategies for reducing water input in irrigated rice; case studies in the Philippines. Agric. Water Manag. 56, 93–112. 10.1016/S0378-3774(02)00007-0

[B32] Tan P. Zeng C. Wan C. Liu Z. Yan M. (2021). Metabolic profiles of *Brassica juncea* roots in response to cadmium stress. Metabolites 11:383. 10.3390/metabo11060383 34199254 PMC8232002

[B33] Tong C. Bao J. (2019). Rice lipids and rice bran oil, in Rice (New York, NY: Elsevier), 131–168. 10.1016/B978-0-12-811508-4.00005-8

[B34] Vega-Gálvez A. Miranda M. Vergara J. Uribe E. Puente L. Martínez E. A. (2010). Nutrition facts and functional potential of quinoa (*Chenopodium quinoa* willd.), an ancient Andean grain: a review. J. Sci. Food Agric. 90, 2541–2547. 10.1002/jsfa.4158 20814881

[B35] Vlachos A. Arvanitoyannis I. S. (2008). A review of rice authenticity/adulteration methods and results. Crit. Rev. Food Sci. Nutr. 48, 553–598. 10.1080/10408390701558175 18568860

[B36] Wu Y.-B. Li G. Zhu Y.-J. Cheng Y.-C. Yang J.-Y. Chen H.-Z.. (2020). Genome-wide identification of QTLs for grain protein content based on genotyping-by-resequencing and verification of qGPC1-1 in rice. Int. J. Mol. Sci. 21:408. 10.3390/ijms21020408 31936451 PMC7014352

[B37] Wurtzel E. T. Kutchan T. M. (2016). Plant metabolism, the diverse chemistry set of the future. Science 353, 1232–1236. 10.1126/science.aad2062 27634523

[B38] Yan N. Du Y. Liu X. Chu M. Shi J. Zhang H.. (2019). A comparative UHPLC-QqQ-MS-based metabolomics approach for evaluating Chinese and North American wild rice. Food Chem. 275, 618–627. 10.1016/j.foodchem.2018.09.153 30724241

[B39] Yang M. Yang J. Su L. Sun K. Li D. Liu Y.. (2019). Metabolic profile analysis and identification of key metabolites during rice seed germination under low-temperature stress. Plant Sci. 289:110282. 10.1016/j.plantsci.2019.110282 31623771

[B40] Yao F. Huang J. Cui K. Nie L. Xiang J. Liu X.. (2012). Agronomic performance of high-yielding rice variety grown under alternate wetting and drying irrigation. Field Crops Res. 126, 16–22. 10.1016/j.fcr.2011.09.018

[B41] Zhang H. Chen T. Wang Z. Yang J. Zhang J. (2010). Involvement of cytokinins in the grain filling of rice under alternate wetting and drying irrigation. J. Exp. Bot. 61, 3719–3733. 10.1093/jxb/erq198 20584789

[B42] Zhang H. Xue Y. Wang Z. Yang J. Zhang J. (2009). An alternate wetting and moderate soil drying regime improves root and shoot growth in rice. Crop Sci. 49, 2246–2260. 10.2135/cropsci2009.02.0099

