# Supplementary figures and images for: Effect of Alternate Wetting and Drying Irrigation on the Nutritional Qualities of Milled Rice
Source: Front Plant Sci. 2021 Sep 9;12:721160. doi: 10.3389/fpls.2021.721160 (PMC8458798; doi:10.3389/fpls.2021.721160)

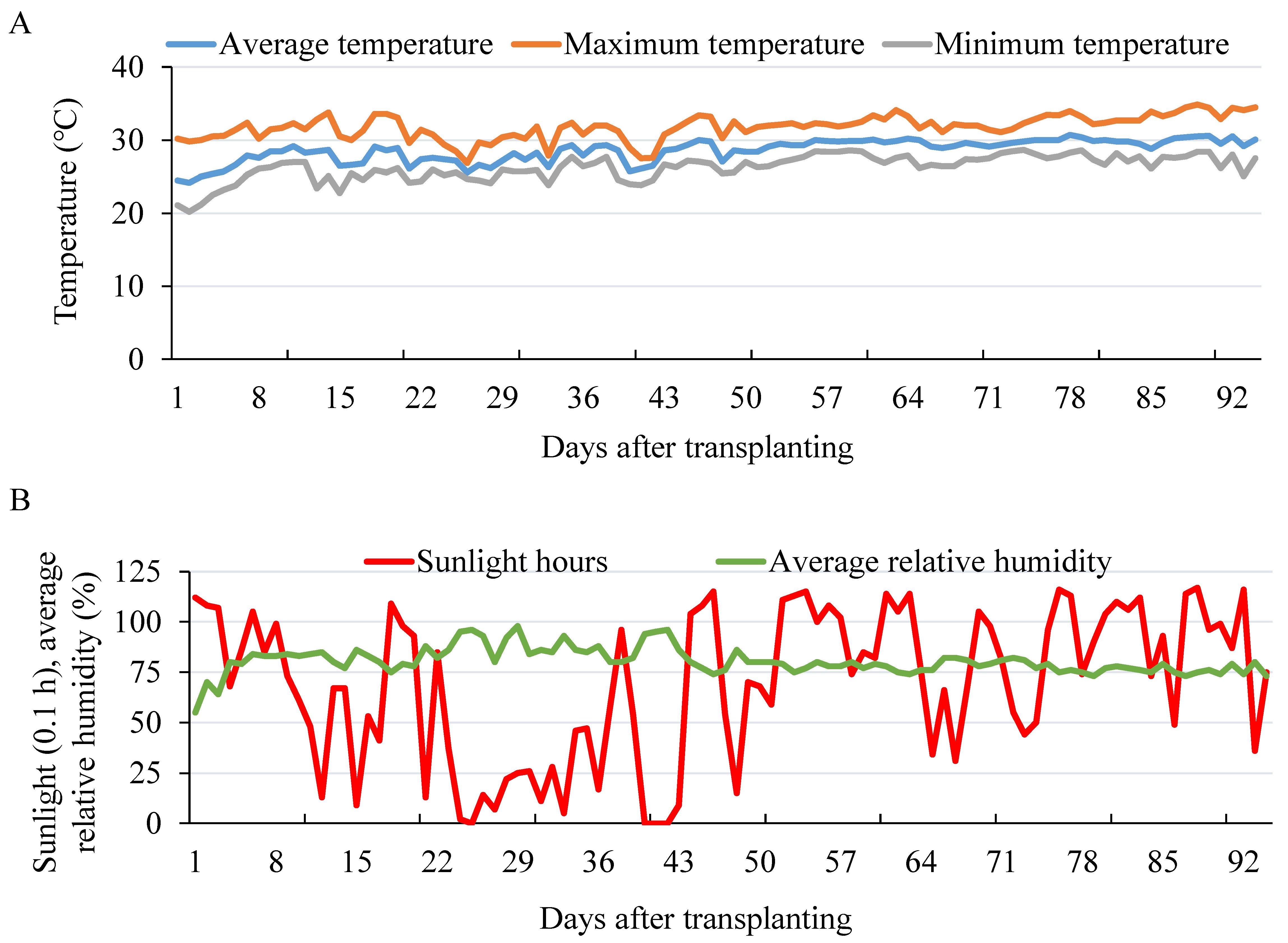


**Fig. S1.** Temperature, sunlight and humidity of greenhouse

Supplement: Supplementary Figure 1 — Temperature, sunlight, and humidity of greenhouse. [file Data_Sheet_2.doc]
